# Supplementary material for: Hybrid Models and Biological Model Reduction with PyDSTool
Source: PLoS Comput Biol. 2012 Aug 9;8(8):e1002628. doi: 10.1371/journal.pcbi.1002628 (PMC3415397; doi:10.1371/journal.pcbi.1002628)
Supplement: Text S4 — Complete source code for the PyDSTool package (version 0.88.120504). Includes API documentation and help files linking to web pages. This file is identical to the current public release on Sourceforge.net. (ZIP) [file pcbi.1002628.s004.zip › PyDSTool/html/PyDSTool.ModelConstructor'.MDescriptor-class.html]

xml version="1.0" encoding="ascii"?


PyDSTool.ModelConstructor'.MDescriptor


| Home | Trees | Indices | Help | PyDSTool |
| --- | --- | --- | --- | --- |

Package PyDSTool ::
Module ModelConstructor' ::
Class MDescriptor

[hide private]

[frames] | no frames]

# Class MDescriptor

source code

```
 object --+        
          |        
common.args --+    
              |    
     Descriptor --+
                  |
                 MDescriptor
```

---

All-in-one descriptor class for hybrid model definitions and
information necessary to be able to build a Model object using a
ModelConstructor call.

generatorspecs should be a dictionary of gen modelspec names ->
modelspecs.


|  |  |
| --- | --- |
| Instance Methods | [hide private] |

|  |  |
| --- | --- |
| add(self, gd) | source code |

|  |  |
| --- | --- |
| get\_desc(self, name) | source code |

|  |  |
| --- | --- |
| isinstantiable(self, verbose=False) | source code |

|  |  |
| --- | --- |
| validate(self) | source code |

**Inherited from `Descriptor`**:
`__init__`,
`__repr__`,
`__str__`

**Inherited from `common.args`**:
`__add__`,
`__cmp__`,
`__contains__`,
`__delitem__`,
`__eq__`,
`__ge__`,
`__getitem__`,
`__gt__`,
`__iter__`,
`__le__`,
`__len__`,
`__lt__`,
`__ne__`,
`__setitem__`,
`clear`,
`copy`,
`fromkeys`,
`get`,
`has_key`,
`info`,
`items`,
`iteritems`,
`iterkeys`,
`itervalues`,
`keys`,
`pop`,
`popitem`,
`setdefault`,
`update`,
`values`

**Inherited from `common.args`** (private):
`_infostr`

**Inherited from `object`**:
`__delattr__`,
`__getattribute__`,
`__hash__`,
`__new__`,
`__reduce__`,
`__reduce_ex__`,
`__setattr__`


|  |  |
| --- | --- |
| Class Variables | [hide private] |

\_checkKeys = `('icvalues', 'parvalues', 'inputs')`

\_defaults = `{'activateAllBounds': False, 'checklevel': 2, 'des...`

\_validKeys = `('changelog', 'orig_name', 'name', 'generatorspec...`


|  |  |
| --- | --- |
| Properties | [hide private] |

**Inherited from `object`**:
`__class__`


|  |  |
| --- | --- |
| Method Details | [hide private] |

|  |  |
| --- | --- |
| validate(self) | source code |

Overrides: Descriptor.validate

  


|  |  |
| --- | --- |
| Class Variable Details | [hide private] |

### \_defaults

Value:
:   ```
    {'activateAllBounds': False,
     'checklevel': 2,
     'description': '',
     'generatorspecs': {},
     'icvalues': {},
     'indepvar': ('t', [-inf, inf]),
     'inputs': {},
     'parvalues': {},
    ...
    ```

### \_validKeys

Value:
:   ```
    ('changelog',
     'orig_name',
     'name',
     'generatorspecs',
     'description',
     'abseps',
     'activateAllBounds',
     'checklevel',
    ...
    ```

  


| Home | Trees | Indices | Help | PyDSTool |
| --- | --- | --- | --- | --- |

|  |  |
| --- | --- |
| Generated by Epydoc 3.0.1 on Fri May 4 15:24:07 2012 | http://epydoc.sourceforge.net |
